# Innovative Milling Processes to Improve the Technological and Nutritional Quality of Parboiled Brown Rice Pasta from Contrasting Amylose Content Cultivars

**DOI:** 10.3390/foods10061316

**Published:** 2021-06-08

**Authors:** Federica Taddei, Elena Galassi, Francesca Nocente, Laura Gazza

**Affiliations:** CREA Research Centre for Engineering and Agro-Food Processing, Via Manziana 30, 00189 Rome, Italy; federica.taddei@crea.gov.it (F.T.); elena.galassi@crea.gov.it (E.G.); francesca.nocente@crea.gov.it (F.N.)

**Keywords:** brown rice, micronization, air fractionation, parboiling, rice pasta

## Abstract

The demand for gluten-free products, including pasta, is increasing and rice pasta accounts for the largest share of this market. Usually, the production of rice pasta requires additives or specific technological processes able to improve its texture, cooking quality, and sensory properties. In this work, two rice cultivars, with different amylose content, were subjected to parboiling, micronization, and flour air fractionation to obtain brown rice pasta, without any supplement but rice itself. In particular, two types of pasta (spaghetti shape) were produced, one from 100% micronized wholemeal, and the other from refined rice flour replaced with 15% of the air-fractionated fine fraction. Regardless of the cultivar, pasta from wholemeal micronized flour showed higher protein and fiber content than refined flour enriched with fine fraction, whereas no differences were revealed in resistant starch and antioxidant capacity. Pasta from the high amylose content genotype showed the highest resistant starch content and the lowest predicted glycemic index along with sensorial characteristics as good as durum semolina pasta in fine fraction enriched pasta. Besides the technological processes, pasta quality was affected the most by the genotype, since pasta obtained from high amylose cv Gladio resulted in the best in terms of technological and sensory quality.

## 1. Introduction

Rice (*Oryza sativa* L.) is the second most important staple food crop, after wheat, and currently sustains half of the world’s population [1]. Indeed, it contains carbohydrates (75–80%), proteins (7–8%), lipids (3%) and is also rich in dietary fiber, minerals, and vitamins, especially when consumed as wholegrain [2].

Owing to the real or presumed increase in gluten intolerances along with changing consumer preferences for more digestible foods, the demand for gluten-free products, including pasta, is increasing [3,4]. Among the gluten-free pasta, currently, rice pasta accounts for a higher value of the gluten-free pasta market share [4], due to its bland flavor, high digestibility, and hypoallergenic properties [5]. Usually, the production of rice pasta requires additives, such as proteins, gums, and emulsifiers, or specific technological process, such as extrusion cooking and hydrothermal treatments, which modify the functional properties of starch and protein, improving texture, cooking quality, and sensory properties of the cooked pasta [6,7].

Rice starch characteristics influence the processability and the technological properties of rice the most [8], mainly the ratio of amylose and amylopectin constituents. Understanding the characteristics of rice starch is very important for optimizing industrial end-products and providing consumers with suitable rice cultivars with enhanced health benefits. Brown rice noodles from high amylose content genotypes exhibited better texture and cooking quality [9]; moreover, starch granules rich in amylose resulted in a more crystalline structure than those with low amylose content. Consequently, they do not swell or gelatinize as readily upon cooking and, therefore, are digested more slowly, resulting in lower blood glucose and insulin responses than low-amylose content rice varieties. For this reason, the intake of high-amylose rice foods has been considered more desirable for individuals with impaired glucose metabolism [10].

Amongst the technological processes usually applied to rice, parboiling is able to modify the physicochemical properties of starch, avoiding the use of additives, such as texturing proteins, gums, and emulsifiers [11]. Indeed, it is reported that parboiling, alters the structural properties of rice starch, leading from crystalline to amorphous form and resulting in the highly compact and translucent endosperm and improving the sensory and cooking qualities, as well as the texture of the rice noodles [12]. Parboiling induces lipid-amylose complexes synthesis, aggregation of soluble proteins, resulting in a reduction of starch swelling and amylose leaching during cooking, in a decrease in stickiness, and in an increase in hardness [13,14]. Upon parboiling, an improvement of nutritional properties of rice also occurs, due to the migration of vitamins and minerals towards the endosperm, together with an increase in the levels of resistant starch (RS) [15,16], that appears to confer considerable benefits to human colonic health [17,18]. The use of flour from parboiled rice as raw material for pasta products [19], allowed to obtain pasta with a good cooking behavior due to the starch arrangements in the product [20].

Despite the higher nutritional value, brown rice is consumed less than white rice because of low consumer acceptability and of problematic technological aspects [21]. Mild separation technologies such as air flow fractionation could be applied for the production of wheat flour fractions, enriched in interesting healthy compounds, such as arabinoxilans, alkylresorcinols, and dietary fiber [22], to be added as ingredients for obtaining cereal-based functional foods, overcoming the technological and sensorial drawbacks of wholegrain. The aim of this work was to evaluate the behavior of two japonica rice cultivars, Gladio and Ronaldo, with contrasting amylose content, subjected to non-conventional transformation processes such as parboiling, micronization, and flour air fractionation to obtain rice pasta with increased nutritional and healthy potential. The effect of both the genotype and the technological process on the nutritional properties of raw materials and pasta samples, cooking quality, and starch hydrolysis index were investigated.

## 2. Materials and Methods

### 2.1. Plant Material

Japonica brown rice kernels (BR) of high amylose cv Gladio and intermediate-low amylose cv Ronaldo, classified according to Juliano [23], were kindly supplied by CREA-Research Centre for Cereal and Industrial Crops (Vercelli, Italy).

### 2.2. Technological Process

**Figure 1:**
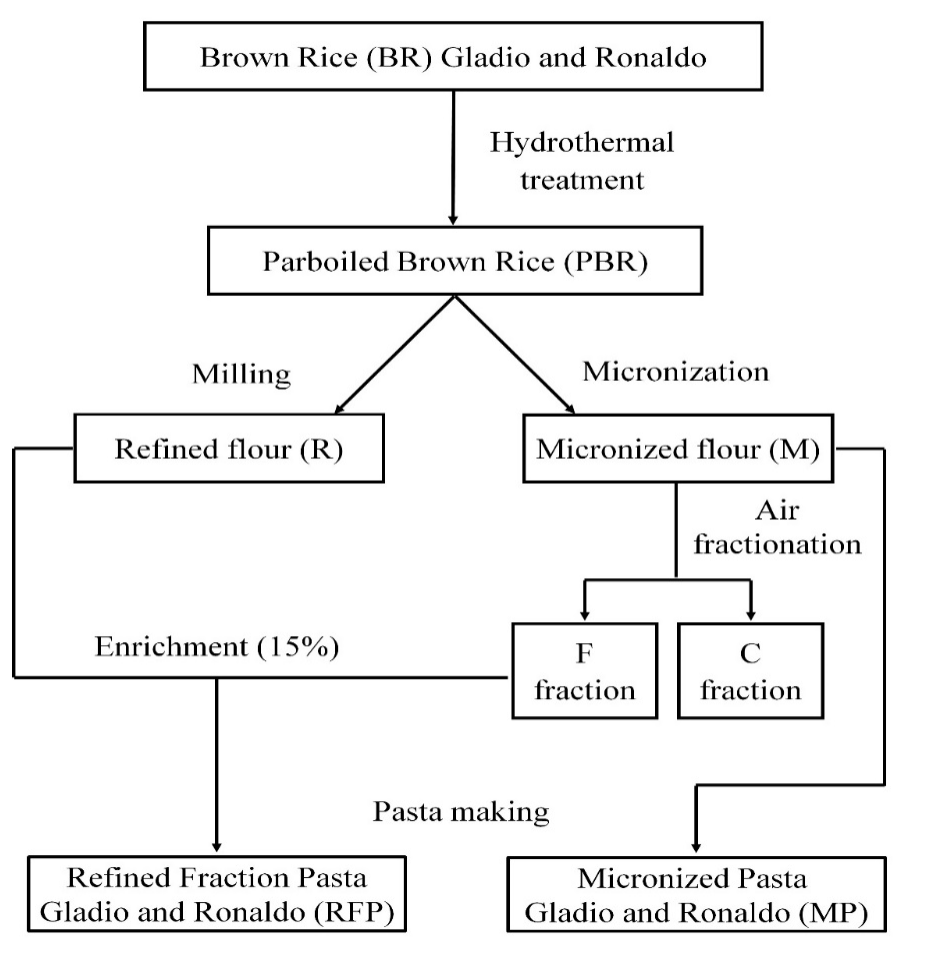
Flow chart of the technological processes applied to the two rice cultivars Gladio and Ronaldo.

The flow chart of the processes applied is represented in Figure 1.

#### 2.2.1. Parboiling Process

Brown rice kernels (BR) of cvs Gladio and Ronaldo were subjected to parboiling process following the method described by Hidalgo et al. [24]. In detail, kernels were conditioned for 4 h, until a moisture content of 15–16% was reached and then heated by steaming at 120 ± 1 °C, 2.1 bar, 10 min. The steamed kernels were dried for almost 48 h to reach 11% moisture, in an oven at 30 °C.

#### 2.2.2. Milling and Ultra-Fine Milling

Parboiled brown rice (PBR) was ground by a milling pilot plant (4RB BONA, Monza, Italy) to obtain refined flour (R). In addition, an aliquot of both parboiled (PBR) and not parboiled brown rice (BR) kernels were ground by the Cyclotec Laboratory Mill (FOSS, Hillerod, Denmark) 1 mm sieving and considered as the reference material. Milling processes were repeated twice. Ultra-fine milling (micronization) was applied on the parboiled kernels, in the KMX-500 device (Separ Microsystem, Brescia, Italy) at a frequency of 170 Hz to obtain micronized flours (M).

#### 2.2.3. Air Fractionation

The micronized flours (M) were fractionated as described in Ciccoritti et al. [22] by a unit integrated turbo air separator (Separ Microsystem, Brescia, Italy) where an aspirating pump drives the air flow, which was modulated setting the inlet restriction valve at 250. The system sorted the flour in two fractions defined as coarse (C) and fine (F).

### 2.3. Pasta Making Process

Two pasta formulations were produced for each cultivar: (i) 100% micronized flour pasta (MP); (ii) 85% refined flour plus 15% F fraction pasta, (RFP) (Figure 1). Tap water was added to obtain a dough with 40% of moisture content. Pasta, spaghetti shape (1.6 mm diameter), was produced using an experimental press (NAMAD, Rome, Italy). Doughs were kneaded for 15 min at 50 °C. Rice pasta samples were dried horizontally by an experimental drier (AFREM, Lyon, France), applying a low temperature drying cycle for 18 h at 50 °C. Pasta from 100% semolina, from durum wheat cultivars Antalis and Svevo, was also produced following the same experimental conditions, except for the dough moisture content which was 34%.

### 2.4. Pasta Color, Cooking Quality and Sensory Test

The color of dried pasta samples was measured by Tristimulus Colorimeter, Chroma Meter CR-400 (Konica Minolta, Osaka, Japan), using the CIE-Lab color space coordinates L* (white-black), a* (red-green) and b* (yellow-blue), and the D65 illuminant (0° viewing angle geometry)

One hundred grams of rice pasta were added to 1L of boiling tap water without salt, according to the AACC method 66–50.01 [25] to obtain cooked rice pasta. Optimum cooking time (OCT) was evaluated according to D’Egidio et al. [26] and determined as when the white central core of the pasta just disappeared when squeezed between two glasses. Water absorption (WA), cooking loss (CL) and sensory analysis by a panel of three trained assessors, were evaluated as reported by Nocente et al. [27]. The sensorial judgment (SJ) was based on three textural characteristics: firmness, stickiness, and bulkiness. Each of the three parameters was evaluated by a score ranging from 10 to 100, by a trained and experienced panel of three assessors. The global value of the sensorial judgment (SJ) was the arithmetic mean of the three textural components [26].

### 2.5. Chemical Characterization

Chemical composition was assessed both on raw materials and dry pasta. Moisture was measured by a thermobalance (Sartorius MA 40, Goettingen, Germany) at 120 °C and all analytical data were expressed as dry weight (dw).

Total starch (TS) content was determined according to the Official Method 996.11 [28], by Total Starch Assay Kit (Megazyme, Bray, Ireland). Amylose content was determined using the Megazyme Amylose/Amylopectin assay kit. Resistant starch (RS) content was determined according to the Official Method 2002.02 [29], using Resistant Starch Assay Kit (Megazyme). Total dietary fiber (TDF) content was measured using the enzymatic kit Bioquant (Merck, Darmstadt, Germany) according to the Official Method 991.42 [30]. Ash content was determined according to the Official Method 08-01.01 [31]. Protein content was determined by micro-Kjeldhal nitrogen analysis, according to the ICC 105-2 method [32], using as conversion factor N × 6.25. Total antioxidant capacity (TAC) was determined according to Martini et al. [33].

#### Starch Hydrolysis Index and Predicted Glycemic Index

Starch hydrolysis was analysed following the method described by Gonĩ et al. [34]. One hundred milligram of cooked pasta samples were homogenized in HCl–KCl buffer pH 1.5 using an Ultra Turrax homogenizer (T25, Ika Labortechnik, Staufen, Germany). Then, samples were digested by pepsin from porcine gastrine mucosa (Merck) followed by α-amylase from porcine pancreas (Merck) and by amyloglucosidase from *Aspergillus niger* (Merck). Glucose concentration was measured using the glucose oxidase–peroxidase (GOPOD) kit (Megazyme). The rate of starch digestion was expressed as a percentage of the total starch hydrolyzed at different times [34]. To describe the kinetics of starch hydrolysis the area under the hydrolysis curve, hydrolysis index and expected glycemic index were estimated using the Goni [34] proposed equations.

### 2.6. Statistical Analysis

All analyses were performed in three replicates unless otherwise stated. Replicated results were expressed as mean ± standard deviation. Means were compared using the Kruskal–Wallis test to highlight significant differences (*p* ≤ 0.05) among the different samples for each considered parameter, followed by Mann–Whitney test for paired comparison of the samples. Principal Component Analysis (PCA) was carried out to investigate the relationships among all pasta variables under study. Software PAST 4.02 (Oslo, Norway) was used to conduct data analysis.

## 3. Results and Discussion

### 3.1. Chemical Characterization of Raw Materials and Milling Products

**Figure 2:**
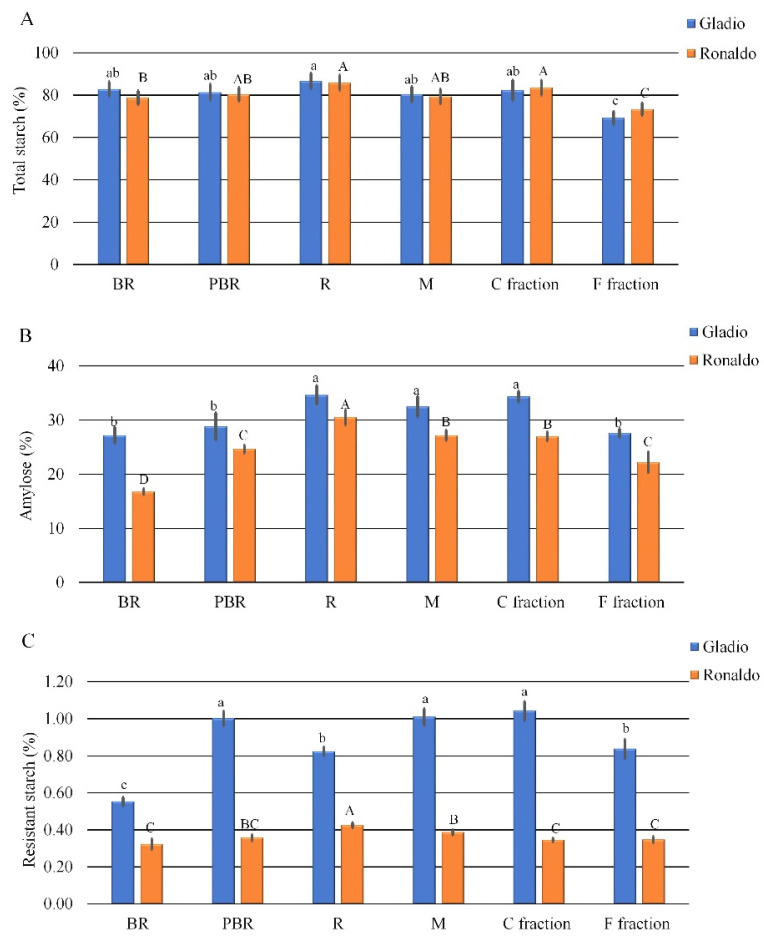
Total starch (**A**), amylose (**B**) and resistant starch (**C**) content in raw materials of rice cultivars Gladio and Ronaldo. BR = Brown Rice; PBR = Parboiled Brown Rice; R = Refined parboiled flour; M = Micronized parboiled flour; C = Coarse; F = Fine. Different letters indicate significant differences determined by the Mann–Whitney pairwise test (*p* ≤ 0.05). Lower case letters refer to cv Gladio; upper case letters refer to cv Ronaldo. Results are expressed as ± standard deviation for three replications.

In both cultivars, brown rice (BR), parboiled brown rice (PBR), and micronized flour (M) showed no significant differences in total starch (TS) content, being the mean value of about 80% (Figure 2A). Refined flour (R) resulted in the highest TS content (86.7 and 85.9% in cv Gladio and Ronaldo, respectively), as a consequence of the outmost layers’ removal leading to an increased contribution of the amylaceous endosperm to the total weight [14]. Air fractionated C fraction presented TS values higher (*p* ≤ 0.05) than F fraction in both cultivars (Figure 2A). This could be explained by the effect of the air fractionation process conditions that led to a major concentration of total starch in the milling fractions presenting flour with higher particle size and lower fiber content, as previously observed also in durum wheat [22].

The analysis of amylose content confirmed the high (cv Gladio) and intermediate-low (cv Ronaldo) amylose trait [35], showing a mean value of 30.8 and 24.6%, respectively (Figure 2B). Parboiling process applied to brown rice kernels determined a general increase in amylose, detectable to a major and significant (*p ≤* 0.05) extent in the intermediate-low amylose cv Ronaldo. This result could be ascribable to the rearrangements of amylose and amylopectin, upon the hydrothermal treatment, as already reported in [20]. In detail, a significant increase in comparison to BR and PBR was observed in refined parboiled flours (R) (34.7% and 30.6% in Gladio and Ronaldo, respectively), devoid of external layers, as above discussed for the TS content. In both cultivars, the C fraction showed a significantly higher amylose percentage than the F fraction (34.4% vs. 27.6% in cv Gladio and 27.0% vs. 22.2% in cv Ronaldo) (Figure 2B), as already observed for the TS. This result could be a consequence of the major structure breakage of starch granules occurring upon micronization mainly in the smallest mean size fraction (F), as also observed by Hossen et al. [36].

Resistant starch content was always significantly higher in cv Gladio than in cv Ronaldo, with a mean value of 0.879% in Gladio and of 0.365% in Ronaldo (Figure 2C). Though the method used [29] could implicate low accuracy (standard error higher than 5%) when applied to samples containing less than 2% resistant starch, it allowed discernment of the differences among all matrices analyzed and specifically between the two cultivars. Results are in accordance with previous findings in which amylose content was found to be positively correlated with resistant starch [35,36,37]. In detail, for parboiled brown rice, RS content increased in both cultivars, with a much higher magnitude in cv Gladio than in cv Ronaldo (+81% vs. +11%, respectively), with a significant increase (*p ≤* 0.05) only in cv Gladio. The observed increase is due to the effect of cooling after the parboiling process that led to starch retrogradation [38,39,40]. Sample R contained less RS than PBR in cv Gladio, whereas in cv Ronaldo, the tendency is the opposite (Figure 2C), likely due to the differences in amylose and the correlated RS content of the two genotypes, which could affect the response to the milling process. On the contrary, micronized flours (M) of both cultivars presented, as expected, RS values similar and not statistically different to those observed in PBR, suggesting that milling method had no effect on RS percentage. The comparison between F and C fractions revealed no significant differences in RS content in cv Ronaldo, but a significant decrease in F fraction was detected in cv Gladio (Figure 2C).

**Figure 3:**
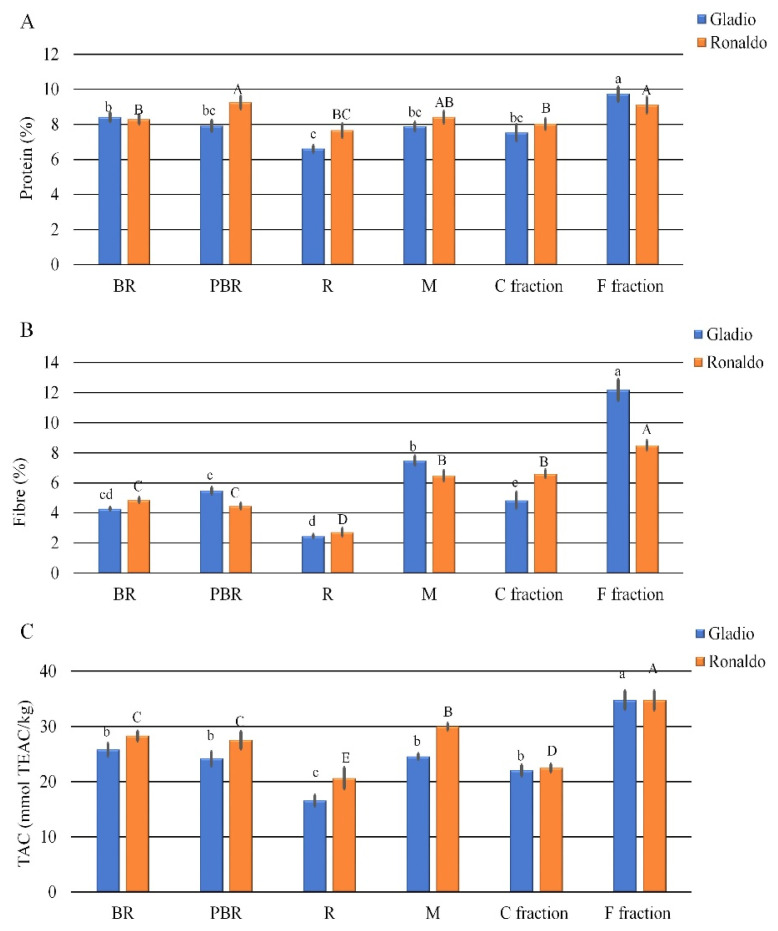
Protein (**A**) and fiber (**B**) content and Total antioxidant capacity (TAC) level (**C**) in raw materials of rice cultivars Gladio and Ronaldo. BR = Brown Rice; PBR = Parboiled Brown Rice; R = Refined parboiled flour; M = Micronized parboiled flour; C = Coarse; F = Fine; TEAC = trolox equivalent antioxidant capacity. Different letters indicate significant differences determined by the Mann–Whitney pairwise test (*p* ≤ 0.05). Lower case letters refer to cv Gladio; upper case letters refer to cv Ronaldo. Results are expressed as ± standard deviation for three replications.

Protein content in brown rice kernels (BR) of both cvs was slightly higher than 8.0% (Figure 3A); after the parboiling process, a slight but not significant decrease in cv Gladio (−6%) and a significant increase in cv Ronaldo (+11.6%) was observed. Though, generally, proteins are reported to be less efficiently extracted from parboiling rice [41], due to leaching, breaking, and gelatinized starch entrapment that occurred over soaking and steaming, some authors [42,43] found a significant increase in the protein content in parboiled rice kernel. This opposite behavior is probably due to the different responses of the genotypes to the parboiling process. Because of the removal of the outer layers, where part of the proteins is located, in the refined flour (R), a not significant decrease in the protein content was observed in cv Gladio (−16.5%), whereas a significant decrease (−17.2%) was detected in cv Ronaldo (Figure 3A). Indeed, in the micronized wholemeal (M), the protein content did not differ from that found in the parboiled kernels (PBR). The air fractionation process led to a protein content significant increase in F fraction with respect to M flour (+23% in Gladio and +8% in Ronaldo, Figure 3A).

Upon parboiling process, only in cv Gladio a slight but not significant fiber content increase was determined in brown rice kernels (Figure 3B). As expected, refined flour (R) showed a significant decrease in fiber content in both cultivars (−55% and −39% in Gladio and Ronaldo, respectively), whereas micronized wholemeal (M) showed a significant increase of 37% in Gladio and of 45% in Ronaldo (Figure 3B). This result could be due to the micronization process which produced a finer flour that could improve the fiber analytical determination, likely because of the increase in the surface area available for the enzyme activity, as already observed about starch [36]. Similar results were reported in micronized wheat [44] and barley flour [45]. The air fractionation process significantly increased the fiber content in both cultivars only in the F fraction (+63% in Gladio and +31% in Ronaldo, Figure 3B), supporting the air fractionation as an eligible technology, able to obtain flour fractions enriched in fibers [22,45] and indicated to improve the nutritional value of rice refined flours that contain low fiber content. However, the air fractionation process was affected by the genotype; indeed, in cv Gladio the major amount of TDF was present in the F fraction, while in cv Ronaldo, fiber was more equally distributed between the two milling fractions (Figure 3B). This result could be due to the different amylose and resistant starch content of the two cultivars which probably affected the textural properties of rice kernel and consequently the particle size of micronized flour.

In both cultivars, the parboiling process caused a small and not significant reduction in TAC levels in brown rice kernels (Figure 3C) because of a certain loss of compounds with antioxidant activity that are sensible to hydrothermal conditions [46]. A further significant decrease was observed in the refined flour (Figure 3C) in which the external layers, where the antioxidant compounds are mostly concentrated, are absent. Micronized wholemeal (M) and parboiled brown rice (PBR) showed similar and not significant differences in TAC levels (Figure 3C) in cv Gladio, whereas a slight but significant increase was observed in cv Ronaldo. As above observed for fiber, proteins, and resistant starch, F fraction showed the highest TAC level (Figure 3C), likely due to the presence of a major content of phenolic acids in the bran [47], indicating this fraction as the richest in bioactive and antioxidant compounds, and therefore, it has been selected to improve the nutritional potential of dry rice pasta. The addition of 15% of F fraction has been valuated as the best compromise in terms of nutritional and sensory texture aspects since this percentage is allowed to reach about 4% of fiber content in pasta formulations, so that they can be defined as a ‘source of fiber’.

### 3.2. Chemical Characterization and Color of Dry Pasta

Since the chemical characteristics of the raw materials include a high level of fiber, resistant starch, TAC and proteins and low total starch content, two pasta samples for each rice cultivar were made, one from 100% micronized wholemeal (MP) and the other from refined rice flour replaced with 15% of the F fraction (RFP). These pasta formulations represented a *unicum* amongst enriched pasta, being obtained by non-conventional technological processes and by enrichment with fractions derived from rice cultivar itself.

Micronized pasta (MP) showed a total starch content slightly but not significantly lower than RFP in both cvs Gladio and Ronaldo (Table 1), due to the presence of the refined flour which mostly contributed (85%) to the RFP pasta formulation. In both cultivars, amylose, and RS content did not statistically differ in MP or in RFP (Table 1). However, it is noteworthy that both pasta samples from cv Gladio exhibited amylose and resistant starch values definitely higher than those revealed in MP and RFP from cv Ronaldo. As previously discussed for raw materials, these results confirmed the positive correlation between amylose and resistant starch content [35,36,37,38].

Statistically significant differences in protein content between MP and RFP pasta were observed only in cv Gladio, being one percentage point lower in RFP than in MP (Table 1). In RFP, the protein content decrease was not as high as expected thanks to the addition of 15% of the F fraction whose protein content was the highest amongst the raw materials (Figure 3A).

Gladio and Ronaldo MP, showed higher fiber content than RFP pasta (+38% and +13%, respectively; Table 1). Nevertheless, the replacement of refined flour with 15% of F fraction allowed to obtain a TDF content as high as 4.5% on average, very similar to the fiber content, 3.0–4.0% usually detected in durum semolina pasta [48].

Both MP and RFP evidenced a low but significant decrease in TAC levels compared to their relative starting raw materials (Table 1); likely the decrement in pasta samples could be explained by the rearrangements occurring in the pasta structure as a consequence of extrusion and drying processes which could affect the accessibility of the ABTS radical to the antioxidant compounds and their thermal degradation, as previously observed in durum wheat pasta by Martini et al. [49]. Noteworthy, in both cultivars, TAC values in RFP samples were very similar (Table 1) to those observed in MP (*p* > 0.05), indicating that the addition of only 15% of F fraction to refined flour determined a remarkable increment of TAC also in refined pasta sample.

As expected in MP, the ash content was higher than in RFP samples (Table 1), even considering that the F fraction gave a great contribution to ash content in RFP value. However, the ash content value, in all four pasta samples, fell within the Italian legal limit for whole semolina pasta (1.8%) [50].

Both pasta formulations from cv Ronaldo showed higher (*p* ≤ 0.05) yellow index (b*) than pasta from cv Gladio (Table 2). Anyway, b* values in all pasta samples fell within the range 23–26 considered as ‘good’ for semolina pasta [51]. These good yellow indices could be mainly due to the parboiling process that allows enhancement of the yellowness of the milled rice kernels because of Maillard reactions and physico-chemical changes in starch and protein components [42,43,52,53]. Hence, all the rice spaghetti produced in the present study, presented good yellow indices, making these pasta formulations inviting even for consumers of durum semolina pasta. Brown (100-L) indices were very similar in all pasta samples, Gladio MP, being the highest (Table 2). However, brown and red values were higher (*p* ≤ 0.05) than those usually obtained from semolina pasta because of the outer layers present in micronized wholemeal pasta (MP) and of the fiber-rich F fraction in RFP, but also as an effect of the parboiling process which is reported to influence brown and red color parameters [53].

### 3.3. Cooking Quality

**Figure 4:**
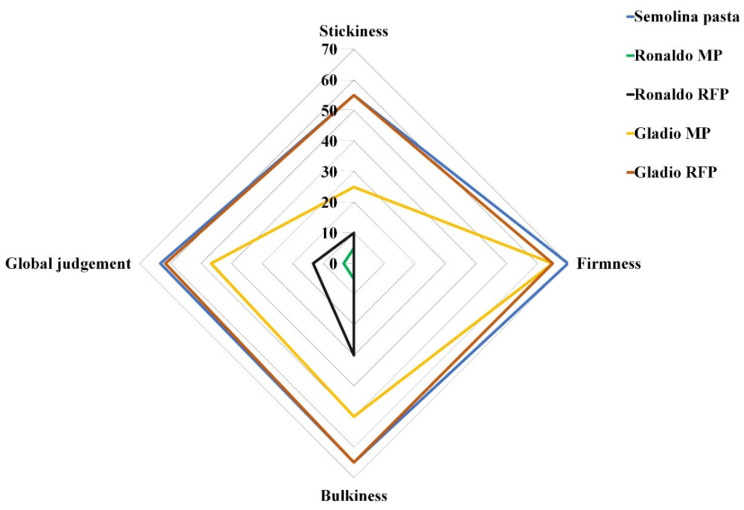
Radar chart of sensory assessment of rice and semolina pasta. MP = Micronized Pasta; RFP = Refined + F Fraction Pasta. For stickiness and bulkiness: ≤20 = very high, >20 and ≤40 = high, >40 and ≤60 = rare, >60 and ≤80 = almost absent, >80 and ≤100 = absent; for firmness, ≤20 = absent, >20 and ≤40 = rare, >40 and ≤60 = sufficient, >60 and ≤80 = good, >80 and ≤100 = very good. Global Sensorial Judgement score ranges from 10 to 100: <55 = scarce, ≥55 and <65 = sufficient, ≥65 and <75 = good, ≥75 = very good.

Rice pasta samples were compared to durum semolina pasta produced in the same pilot plant at the same extrusion and drying conditions. In both rice cultivars, the optimal cooking time (OCT) was significantly higher in MP, than in RFP (Table 2). The absence of gluten and the presence of the fiber might make the water absorption easier, since fiber has higher water absorption than gluten proteins, hence reducing the cooking time with respect to traditional semolina pasta. Indeed, a significant decrement was observed for water absorption (WA) in all pasta samples in comparison to semolina pasta (Table 2). Moreover, in both cultivars, MP adsorbed almost half the amount of water with respect to RFP. These results are in accordance with findings already observed in other bran-enriched pasta [27,54,55], since the fiber retains less water with respect to the starch. Through the parboiling process, this was demonstrated to induce lipid-amylose complex reducing starch swelling and amylose leaching during cooking [13], the absence of gluten caused, in all rice pasta formulations, a cooking loss heavy higher than in semolina pasta (Table 2), as already observed by Kaur et al. [56]. The global sensorial judgment (Figure 4), focused on the sensory texture quality traits, revealed that Gladio was the most suitable cultivar for rice pasta formulation, the RFP reaching values as good as those of semolina pasta, with the consensus of all of the three experienced assessors. Noteworthy, in both cultivars, the enrichment with F fraction improved both stickiness and bulkiness sensorial parameters whereas no effect was observed on firmness. This last parameter was instead mostly affected by the amylose (*r*^2^ = 0.91) and resistant starch content (*r*^2^ = 0.80) [14], which resulted higher in cv Gladio, hence improving rice spaghetti firmness. The observed huge differences between pasta from cv Gladio and cv Ronaldo highlighted the importance of the genotype choice to obtain products with suitable characteristics that could meet the rice pasta consumers’ acceptance.

### 3.4. Starch Hydrolysis and Predicted Glycemic Index

Results obtained from the in vitro method for the determination of starch hydrolysis revealed that all rice pasta presented hydrolysis indices (HI) lower (*p* ≤ 0.05) than semolina pasta, high amylose Gladio pasta samples showing lower (*p* ≤ 0.05) values than Ronaldo ones (Table 3). It could be inferred that the parboiling process coupled with the presence of fiber, exerted a crucial role in lowering the starch hydrolysis and consequently the glycemic index (GI). Indeed, as already reported by Zohoun et al. [16], starch hydrolysis is more efficient for low than for high amylose rice cultivars resulting in a lower glycemic response. Moreover, the formation of resistant starch upon rice parboiling, reduced starch digestibility and glycemic index [57], because of crystallization of amylose after hydrothermal treatment, which changes starch accessibility by hydrolytic enzymes. The differences in HI and GI observed between the two varieties, indicated that the employment of a specific genotype [58] coupled with an appropriate technological process, could allow to obtain rice pasta with lower glucose release.

**Figure 5:**
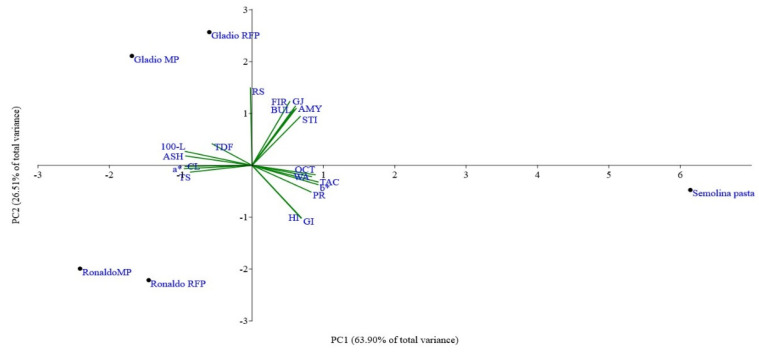
PCA biplot of descriptive analysis of sensory texture parameters: firmness (FIR), bulkiness (BUL), stickiness (STI), global sensorial judgment (GJ); of technological and nutritional parameters: yellow (b*), brown (100-L) and red (a*) indices, water absorption (WA), cooking loss (CL), optimal cooking time (OCT), ash (ASH), total starch (TS), resistant starch (RS), amylose (AMY), protein content (PR), total dietary fiber (TDF), total antioxidant capacity (TAC), hydrolysis (HI), and glycemic (GI) indices, detected in semolina pasta, in micronized pasta (MP) and in refined + F fraction pasta (RFP) from rice cvs Gladio and Ronaldo.

Summing up, the biplot of PCA (Figure 5), obtained by combining all pasta sample variables, allowed to distinguish three main groups, Gladio and Ronaldo rice pasta in the second and third quadrants, respectively, whereas semolina pasta in the fourth one. Moreover, rice pasta occurred in two subpopulations attributed to the different genotypes which were discriminated in relation to amylose and resistant starch as well as in the three sensory texture parameters (bulkiness, stickiness, and firmness). The first two principal components (PC1 and PC2, Figure 5) accounted for 63.9% and 26.5% of the total variance, respectively. The first component was positively associated mainly with protein content, TAC level, b* value, OCT, and WA and negatively with ash and TS content, CL, brown and red indices. The second component was mostly associated with RS and amylose content, and sensory texture parameters and negatively with HI and GI.

## 4. Conclusions

In this study, two rice cultivars differing in amylose content were evaluated to produce brown rice spaghetti obtained from parboiled micronized or refined flour-enriched with air classified fine fraction. The addition of this flour fraction allowed to obtain rice pasta (RFP) with superior sensorial performances than wholegrain pasta (MP), with comparable nutritional properties, by the exclusive use of rice itself. Besides the technological processes, pasta quality was affected the most by the genotype, since pasta obtained from high amylose cv Gladio resulted in the best in terms of nutritional, technological, and sensorial quality. Indeed, pasta from Gladio showed the highest RS content and the lowest glycemic index along with sensorial characteristics as good as durum semolina pasta. Further studies should be addressed to investigate the behavior of additional rice cultivars with contrasting amylose content in terms of pasta making attitude, in order to confirm the role of amylose and resistant starch in pasta quality. Finally, in vivo test should be conducted with the aim to estimate the post-prandial blood glucose content upon the ingestion of differently processed rice pasta products.

## Figures and Tables

**Table 1:** Basic composition and total antioxidant capacity of pasta.

	TS	Amylose	RS	Protein	TDF	TAC	Ash
	(%)	(%)	(%)	(%)	(%)	(mmol TEAC/kg)	(%)
Gladio MP	83 ± 0.7 ^ab^	18.3 ± 0.9 ^a^	0.7 ± 0.03 ^a^	8.86 ± 0.06 ^b^	6.2 ± 0.1 ^a^	21.7 ± 0.3 ^c^	1.59 ± 0.01 ^a^
Gladio RFP	84.7 ± 0.3 ^a^	17.8 ± 0.4 ^a^	0.78 ± 0.03 ^a^	7.73 ± 0.04 ^c^	4.49 ± 0.04 ^b^	22.0 ± 0.2 ^c^	1.29 ± 0.01 ^bc^
Ronaldo MP	84.2 ± 0.6 ^a^	12.8 ± 0.4 ^b^	0.22 ± 0.03 ^c^	8.94 ± 0.07 ^b^	5.2 ± 0.1 ^ab^	24.4 ± 0.1 ^b^	1.47 ± 0.01 ^b^
Ronaldo RFP	85.9 ± 0.6 ^a^	12.2 ± 0.4 ^b^	0.19 ± 0.03 ^c^	8.87 ± 0.04 ^b^	4.6 ± 0.1 ^b^	23.6 ± 0.3 ^bc^	1.36 ± 0.01 ^b^
Semolina pasta	78.3 ± 0.3 ^b^	19.6 ± 0.7 ^a^	0.38 ± 0.01 ^b^	11.55 ± 0.03 ^a^	4.2 ± 0.3 ^b^	46.8 ± 0.6 ^a^	0.71 ± 0.01 ^c^

Results are expressed as mean ± standard deviation. Values with different letters within the same column indicate significant differences determined by the pairwise Mann–Whitney test (*p* ≤ 0.05). MP = Micronized Pasta; RFP = Refined + F Fraction Pasta; TS = total starch; RS = resistant starch; TDF = total dietary fiber; TAC = total antioxidant capacity; TEAC = trolox equivalent antioxidant capacity.

**Table 2:** Color and cooking properties of rice and semolina pasta.

	Yellow index (b*)	Brown Index (100-L)	Red Index (a*)	OCT (min′ s′′)	WA (g)	Cooking Loss (%)
Gladio MP	23.5 ± 0.4 ^c^	58.4 ± 0.9 ^a^	5.33 ± 0.07 ^bc^	8′50′′ ± 5′′ ^b^	54.9 ± 0.2 ^d^	2.40 ± 0.01 ^b^
Gladio RFP	24.3 ± 0.4 ^c^	55.3 ± 0.2 ^b^	4.5 ± 0.1 ^d^	8′15′′ ± 5′′ ^c^	96.9 ± 0.2 ^c^	2.42 ± 0.02 ^b^
Ronaldo MP	25.8 ± 0.3 ^b^	56 ± 1 ^b^	5.7 ± 0.3 ^b^	8′50′′ ± 5′′ ^b^	55.6 ± 0.2 ^d^	3.096 ± 0.016 ^a^
Ronaldo RFP	25.9 ± 0.3 ^b^	55.8 ± 0.4 ^b^	6.0 ± 0.1 ^a^	8′15′′ ± 5′′ ^c^	105.9 ± 0.3 ^b^	2.29 ± 0.02 ^c^
Semolina pasta	40.8 ± 0.2 ^a^	41.5 ± 0.4 ^c^	1.47 ± 0.08 ^e^	10′30′′ ± 5′′ ^a^	148.6 ± 0.5 ^a^	0.367 ± 0.003 ^d^

Results are expressed as mean ± standard deviation. Values with different letters within the same column indicate significant differences determined by the pairwise Mann–Whitney test (*p* ≤ 0.05). MP = Micronized Pasta; RFP = Refined + F Fraction Pasta; OCT = optimal cooking time; WA = water absorption.

**Table 3:** Hydrolysis and predicted glycemic index of rice and semolina pasta.

	HI	GI
Gladio MP	46.10.4 ^d^	65.00.2 ^c^
Gladio RFP	471 ^d^	65.30.6 ^c^
Ronaldo MP	591.08 ^c^	72.20.6 ^b^
Ronaldo RFP	703 ^b^	782 ^ab^
Semolina pasta	82.00.6 ^a^	84.70.3 ^a^

Results are expressed as mean ± standard deviation. Values with different letters within the same column indicate significant difference determined by the pairwise Mann–Whitney test (*p* ≤ 0.05). MP = Micronized Pasta; RFP = Refined + F Fraction Pasta; HI = hydrolysis index; GI = glycemic index.

## Data Availability

Data are contained within the article.
